# Two-Dimensional Platinum Diselenide: Synthesis, Emerging Applications, and Future Challenges

**DOI:** 10.1007/s40820-020-00515-0

**Published:** 2020-08-27

**Authors:** Youning Gong, Zhitao Lin, Yue-Xing Chen, Qasim Khan, Cong Wang, Bin Zhang, Guohui Nie, Ni Xie, Delong Li

**Affiliations:** 1 Institute of Microscale Optoelectronics, College of Physics and Optoelectronic Engineering, Shenzhen University, Shenzhen, 518060 People’s Republic of China; 2 Faculty of Information Technology, Macau University of Science and Technology, Macau, 519020 People’s Republic of China; 3 Department of Mechanical and Mechatronics Engineering, University of Waterloo, Waterloo, ON Canada; 4 Otolaryngology Department and Biobank of the First Affiliated Hospital, Shenzhen Second People’s Hospital, Health Science Center, Shenzhen University, Shenzhen, 518060 People’s Republic of China

**Keywords:** Platinum diselenide, Two-dimensional materials, Photodetector, Photocatalytic, Photoelectric

## Abstract

In recent years, emerging two-dimensional (2D) platinum diselenide (PtSe_2_) has quickly attracted the attention of the research community due to its novel physical and chemical properties. For the past few years, increasing research achievements on 2D PtSe_2_ have been reported toward the fundamental science and various potential applications of PtSe_2_. In this review, the properties and structure characteristics of 2D PtSe_2_ are discussed at first. Then, the recent advances in synthesis of PtSe_2_ as well as their applications are reviewed. At last, potential perspectives in exploring the application of 2D PtSe_2_ are reviewed.

## Highlights

- A comprehensive review of the recent development of two-dimensional (2D) PtSe_2_ synthesis strategies has been extensively surveyed.
- The applications of 2D PtSe_2_ materials in areas, including opto/electric devices, photocatalysis, hydrogen evolution reaction, and sensors, have been reviewed.
- Current challenges in the development of 2D PtSe_2_ materials are identified, and outlooks toward unexplored research areas are suggested.

## Graphical abstract

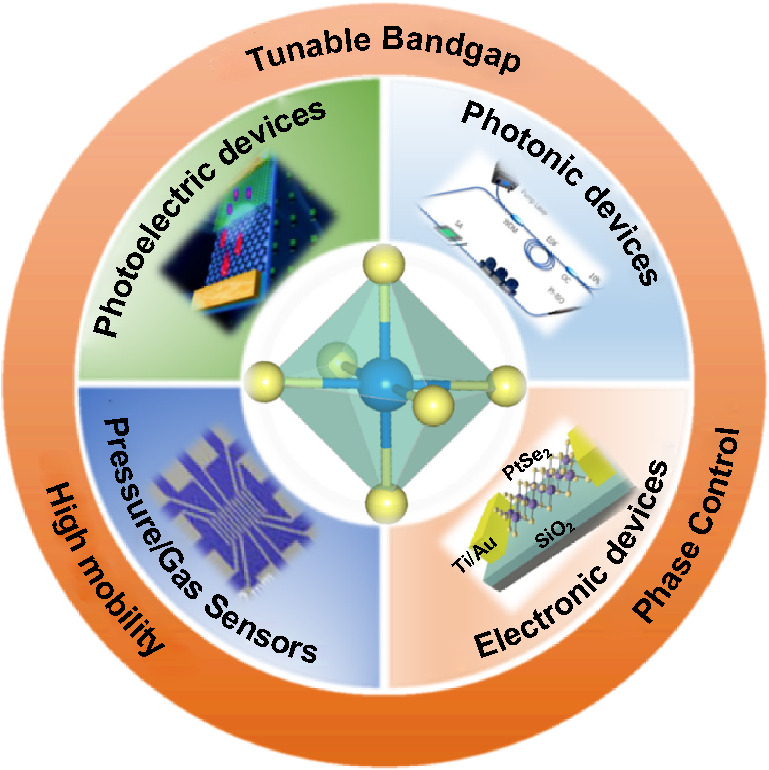

## Introduction

Since graphene was discovered in 2004 [1], two-dimensional (2D) materials have attracted extensive attention due to their unique structure and outstanding properties [2–7]. Recently, layered 2D transition metal dichalcogenides (TMDCs) materials have become one of the hottest research topics due to a large potential in future nanoelectronics [8–15]. Unique physical phenomenon confining the transport of charge and heat in unique layered structure, which are not easily observed or measured in the related bulk crystal, has endowed them an attractive and promising 2D material for electronic, optoelectronic, and spintronic applications [16–19]. Different from the zero-band gap of graphene, TMDCs with tunable finite band gap and significant transitional behavior are more suitable for fabricating high-performance electronic and optoelectronic devices. In the last decades, group-6 TMDCs (such as MoS_2_, MoSe_2_, MoTe_2_, WS_2_, and WSe_2_) which occur naturally in the 2H phase have attracted the most attention [18–22]. However, group-10 TMDCs (such as PtSe_2_, PtS_2_, PdSe_2_, and PdS_2_) which occur naturally in the 1T phase have been theoretically predicted as an outstanding material [23–30]. In addition, experimentally demonstrated distinct properties of group-10 TMDCs have made it prominent than other state-of-the-art 2D materials.

Among 2D group-10 noble TMDCs materials, platinum diselenide (PtSe_2_) has emerged as promising materials for investigating quasiparticle interactions and for developing photoelectric devices [31–33]. Single-layer and few-layer PtSe_2_ are *p*-type semiconductors, and thicker PtSe_2_ exhibit typical semimetallic characteristics [27, 34]. Recently, due to their outstanding properties including widely tunable band gap, high carrier mobility, and excellent air stability, PtSe_2_ has become increasingly fascinating in the 2D materials research [34–37]. 2D PtSe_2_ has exhibited potential in many areas such as photocatalytic, hydrogen evolution reaction, electronic, and optoelectronic devices [38–40].

As an emerging 2D material, PtSe_2_ possesses not only the merits of previously discussed 2D materials, but also many unique advantages. For examples, PtSe_2_ exhibits a strong layer-dependent band structure. Bulk PtSe_2_ exhibits semimetallic character, while monolayer and few-layer PtSe_2_ are semiconductors [25, 41–43]. Moreover, PtSe_2_ exhibits anisotropic carrier mobility along different directions. The theoretically calculated carrier mobility of PtSe_2_ is larger than 3250 cm^2^ V^−1^S^−1^ (*x* direction) and 16,300 cm^2^ V^−1^S^−1^ (*y* direction) at room temperature, respectively [28]. The theoretically predicted carrier mobility is at least 8 times larger than that of MoS_2_ (about 410 cm^2^ V^−1^S^−1^ for *x* direction and 430 cm^2^ V^−1^S^−1^ for *y* direction) [28]. The outstanding inherent properties (including tunable band gap and carrier mobility) of PtSe_2_ are comparable to black phosphorus (BP), but the stability of PtSe_2_ is much better than BP [44–46]. Besides, experimental and theoretical studies have proven the intriguing transport properties and interesting spin physics of PtSe_2_. Overall, these outstanding properties motivating further studies of the electrical transport properties, optoelectronic properties, and piezo-resistivity of 2D PtSe_2_.

Herein, we divulge a comprehensive review based on experimental and theoretical research evolution on 2D layered PtSe_2_, covering the progress, challenges, and prospects in future 2D material. The crystal structure, electronic band structure, and properties of few-layer PtSe_2_ are introduced to give an overview of this material. Next, some recent progress on the various methods to synthesis monolayer and few-layer PtSe_2_, including mechanical exfoliation, chemical vapor deposition (CVD), thermally assisted conversion (TAC), molecular beam epitaxy (MBE), and chemical vapor transport (CVT), are discussed in detail. Furthermore, the applications of 2D PtSe_2_ in many areas, including photodetector, field effect transistors (FETs), mode-locked laser, photocatalytic, hydrogen evolution reaction (HER), and sensors, are highlighted. At last, the perspectives and outlooks for the 2D PtSe_2_ materials are concluded.

## Structure of 2D PtSe_2_

### Crystal Structure

Generally, there are two common structural phases for monolayer TMDCs, which are characterized by either octahedral trigonal prismatic (2H or D_3h_) or (1T or D_3d_). Unlike group-6 TMDCs, group-10 TMDCs tend to form *d2sp3* hybridization due to group-10 metal atoms hold rich *d*-electrons and less *d* orbitals are involved. As a result, group-10 TMDCs lead to the generation of the thermodynamically favored 1T-phase. The 2D layered structure of TMDCs (such as PtS_2_/PtSe_2_/PtTe_2_ and PdS_2_/PdSe_2_/PdTe_2_) has been proposed in 1950s since the pioneering work of Kjekus et al. and Grønvold et al. [47–49]. As a rising star of group-10 TMDCs, PtSe_2_ has a thermodynamically favored 1T-phase structure and the atoms stack in the AA arrangement [28, 50].

**Fig. 1:**
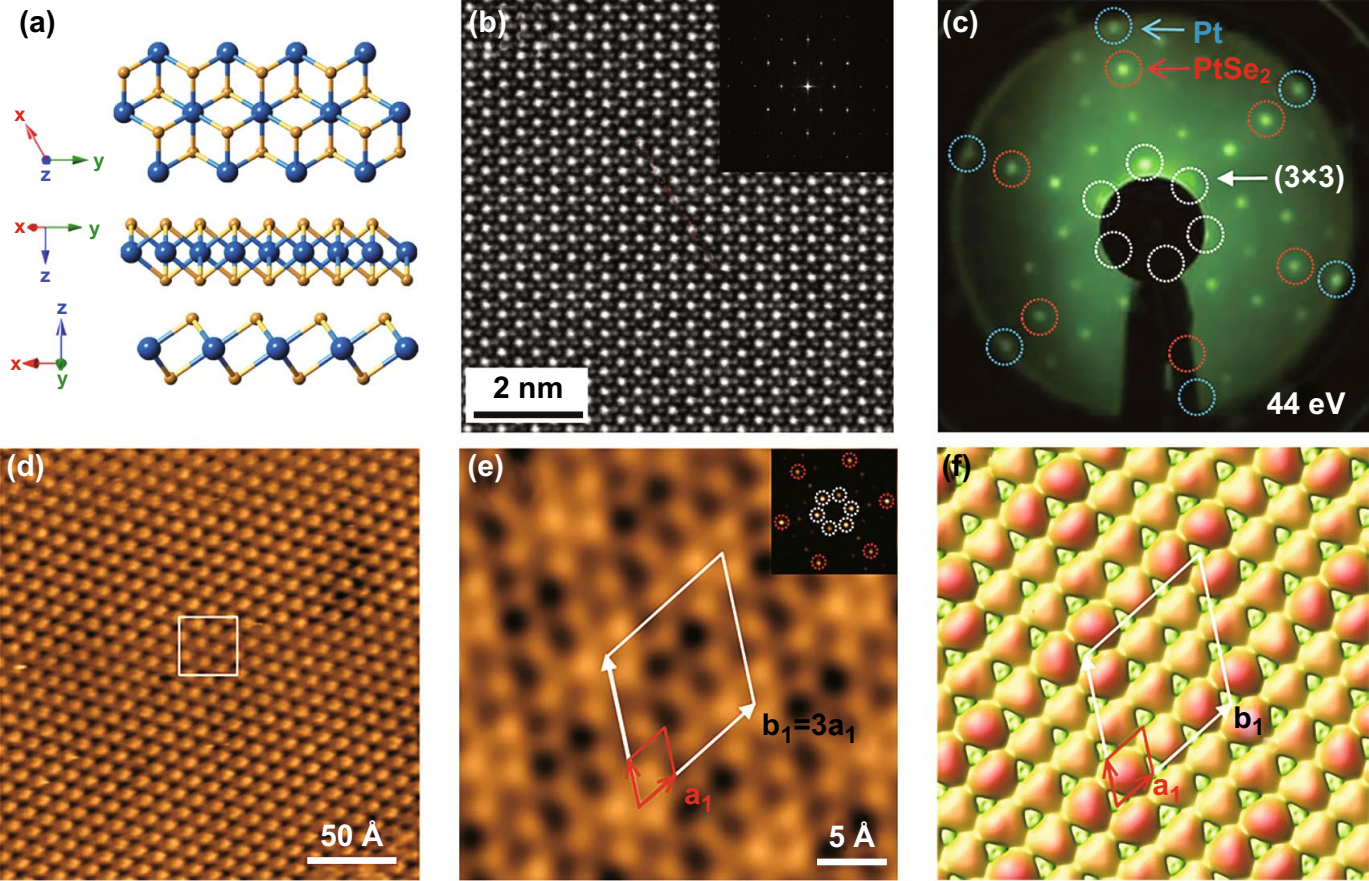
Simulated and characterized crystal structure of PtSe_2_. **a** Crystal structure of PtSe_2_ from different view. The gold balls represent Se atoms and blue balls represent Pt atoms. Reproduced with permission [33]. Copyright 2019, Springer Nature. **b** HR-STEM image of a few-layer PtSe_2_, inset: fast Fourier transform of the image. Reproduced with permission [28]. Copyright 2018, John Wiley and Sons. **c** LEED pattern of a monolayer PtSe_2_ film on Pt substrate. **d** Large scale and **e** atomic resolution STM image of monolayer PtSe_2_ film. **f** Simulated STM image of PtSe_2_ by DFT calculation. Reproduced with permission [25]. Copyright 2015, American Chemical Society

PtSe_2_ crystal belongs to the *D*_*3d*_^*3*^*(P3m1)* space group of the trigonal system [34, 51, 52]. The crystal structure of PtSe_2_ from different view is shown in Fig. 1a. Many techniques have been employed to characterize the atomic structure of monolayer PtSe_2_, such as high-resolution scanning transmission electron microscope (HR-STEM), scanning tunneling microscope (STM), low energy electron diffraction (LEED), and density functional theory (DFT) calculation. As shown in Fig. 1b–f, the HR-STEM image, LEED patterns, STM images, and simulated STM images are presented, respectively. Figure 1b shows the representative HR-STEM image of PtSe_2_. The fast Fourier transform of the image (inset of Fig. 1b) shows hexagonal structure and confirms the single-crystalline feature of the few-layer PtSe_2_ samples. The HR-STEM image of PtSe_2_ clearly shows that each Pt atom is in a tilted octahedral site and surrounded by six Se atoms, which is consistent with the octahedral structure of 1T phase TMDCs [28, 53, 54].

As shown in Fig. 1c, hexagonal diffraction spots from monolayer PtSe_2_ film are observed in a LEED pattern. The STM image and enlarged atomic resolution image of monolayer PtSe_2_ are shown in Fig. 1d, e. By employing LEED, STEM, and STM methods, the atomic structure of PtSe_2_ and lattice constant (*a*_1_ = 3.7 Å, shown in Fig. 1e) are experimentally defined. Moreover, Wang et al. [25] conducted the DFT simulation based on the structure parameters obtained from the experimental characterizations. The simulated STM image is shown in Fig. 1f, and the results are well consistent with the STM observation results, which strongly demonstrated the highly crystalline structure of the 2D layered PtSe_2_.

### Electronic Band Structure

**Fig. 2:**
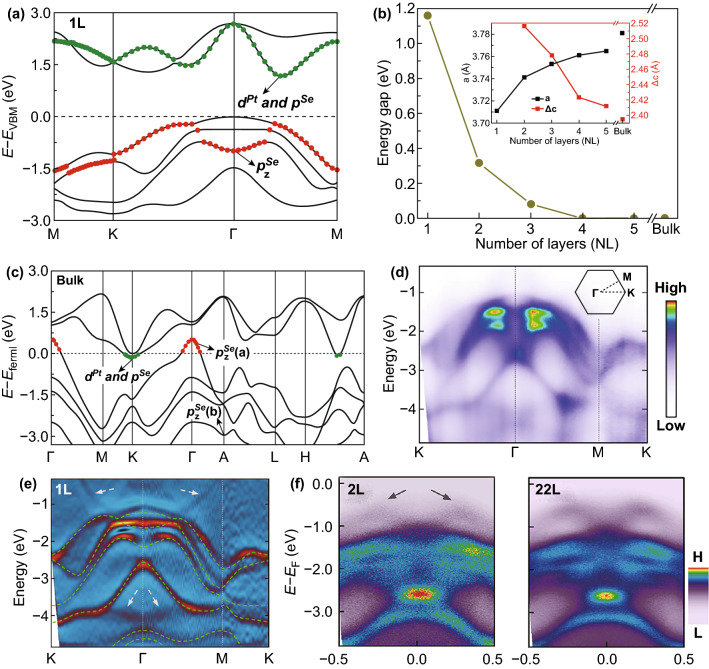
Electronic structure of PtSe_2_. **a** Band structure of monolayer PtSe_2_. Reproduced with permission [28]. Copyright 2018, John Wiley and Sons. **b** Energy gap evolution as a function of number of layers (NL) for PtSe_2_. Inset: layer dependence of lattice constant versus NL. Reproduced with permission [27]. Copyright 2019, Elsevier. **c** Band structure of bulk PtSe_2_. Reproduced with permission [28]. Copyright 2018, John Wiley and Sons. **d** ARPES spectra and **e** corresponding second derivative spectra of monolayer PtSe_2_. Reproduced with permission [25]. Copyright 2015, American Chemical Society. **f** ARPES spectra of bilayer and 22-L PtSe_2_. Reproduced with permission [43]. Copyright 2015, American Chemical Society

The electronic structure of 2D layered TMDCs materials strongly depends on the coordination environment of transition metal and its *d* electron count [23]. PtSe_2_ presents a layer-dependent band structure with dimensional reduction from bulk to monolayer. Zhao et al. [28] found that the monolayer PtSe_2_ is an indirect semiconductor and the band gap of monolayer PtSe_2_ is about 1.17 eV. Figure 2a shows the band structure of monolayer PtSe_2_. The valence band maximum (VBM) of monolayer PtSe_2_ situated at the Г point, which comprised of the *p*_*x*_ and *p*_*y*_ orbitals of Se atoms (*p*^*Se*^_*x* and *y*_). The conduction band minimum (CBM) of monolayer PtSe_2_ is situated between the Г and M points, which is dominated by *d* states of Pt and *p* states of Se. The band gap of PtSe_2_ abruptly decreased with the increased number of layers (NL), due to the exceptionally strong interlayer electronic hybridization of *p*_*z*_ orbital of Se atom (*p*^*Se*^_*z*_).

Figure 2b presents the band gap evolution of PtSe_2_ as function of NL. The band gap of PtSe_2_ shows a sharp decrease as the NL increased. As the NL larger than four the PtSe_2_ shows a semiconductor-to-metallic transition. With increase in stacked layers, the energy level of VBM exceeds that of CBM between Г and M because of the increase in interlayer electronic hybridization [23, 28]. As a result, a semiconductor-to-semimetal evolution occurred. It has been proved that thicker PtSe_2_ (layer numbers large than four or five) becomes semimetallic without a band gap [25, 28, 55]. As shown in Fig. 2c, the band gap structure of bulk PtSe_2_ explicitly shows the semimetallic characteristics, and the CBM moves from a point between the Г and M to the K point due to the strong interlayer interaction of PtSe_2_ [28].

In 2015, Wang et al. [25] experimentally measured the band structure of monolayer PtSe_2_ for the first time by using angle-resolved photoemission spectroscopy (ARPES). Figure 2d shows the ARPES spectra data measured along the high symmetry direction K–Γ–M–K in the hexagonal Brillouin zone at photon energy of 21.2 eV. As shown in Fig. 2e, the location of VBM and CBM in the derivative spectra indicates that monolayer PtSe_2_ is a semiconductor. The ARPES results show excellent quantitative agreement with the DFT simulation results. With this pioneer work, ARPES has become one of the most important techniques to investigate the electronic structure of PtSe_2_ [43, 56, 57]. In order to study the layer-dependent electronic structure, Yan et al. measured ARPES data along the Γ–K direction [43]. As indicated by gray arrow in Fig. 2f, an M shape band was observed in thicker PtSe_2_ (NL ≥ 2). Moreover, the M-shaped band moves toward the Fermi energy as the atomic layers increased, indicating a reduction of the band gap. Therefore, the ARPES results provide direct evidences for the layer-dependent band gap of PtSe_2_ as theoretically predicated [27, 58, 59].

## Properties of 2D PtSe_2_

This section highlights the unique properties of 2D PtSe_2_ such as band gap tenability, phase transition, and vibration spectroscopic and optical properties. The band gap tuned by various kinds of external parameters has been introduced at first. Then, the phase transition of 1T phase, 1H phase, and non-layered PtSe_2_ are reviewed. At last, the vibration spectroscopic and optical properties are introduced in details.

### Band Gap Tunability

**Fig. 3:**
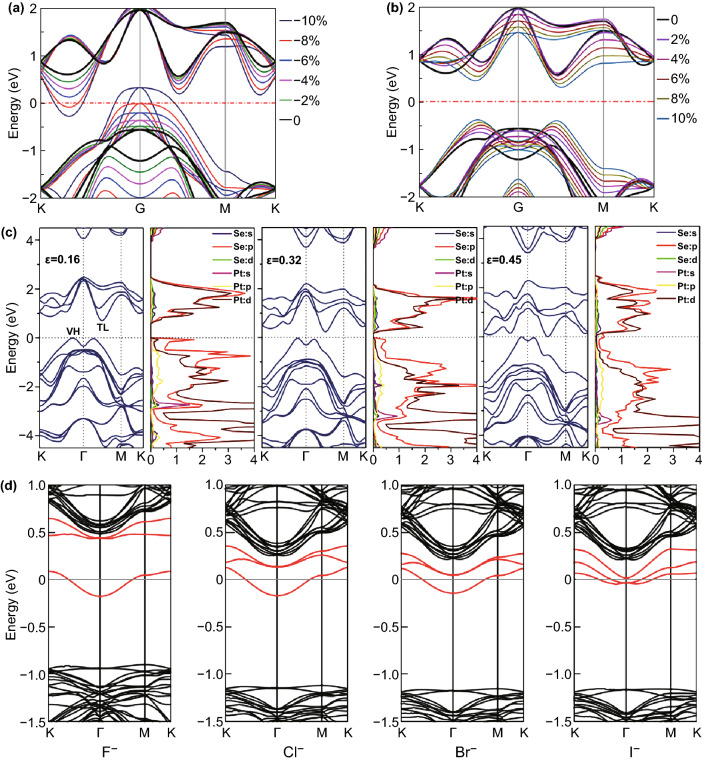
Band structure engineering of PtSe_2_. Computed band structure of the monolayer PtSe_2_ with symmetrical biaxial compressive (**a**) and tensile strains (**b**). Reproduced with permission [65]. Copyright 2018, American Chemical Society. **c** Band structure and density of state of bilayer PtSe_2_ under vertical compressive strain (*ε* = 0.16, 0.32, and 0.45). Reproduced with permission [52]. Copyright 2016, American Chemical Society. **d** Computed band structure of the F-doped, Cl-doped, Br-doped, and I-doped monolayer PtSe_2_. Reproduced with permission [36]. Copyright 2018, American Physical Society

It has been widely proved that the band structure of 2D TMDCs can be tuned by doping, defect engineering, strain, and external electric field [60–64]. Besides the inherent thickness-dependent band gap, band gap of PtSe_2_ also can be tuned by applying external parameters. For example, band gap of few-layer PtSe_2_ can be tuned over a wide range by applying strain. The band structure of monolayer PtSe_2_ with symmetrical biaxial compressive strains and symmetrical tensile strains reveals the band structure evolution, as shown in Fig. 3a, b [65]. Du et al. [66] have also demonstrated that the band gap decreases approximately linearly with the increased tensile strain, but it is different for the band gaps evolution under compressive strain. As shown in Fig. 3a, monolayer PtSe_2_ exhibits a direct gap semiconductor characteristic as the compressive strain reaches − 8%. The same transformation has also been reported by other published papers [52, 66–68]. Moreover, due to the chemical interaction (*p* orbital coupling) between Se atoms of the two layers, a reversible semiconducting metallic transition bilayer PtSe_2_ under critical vertical strain, as shown in Fig. 3c [52]. Besides strain engineering, doping also has significant effect on the band structure of 2D PtSe_2_ [36, 39, 69]. As shown in Fig. 3d, the band structures of the halogen elements (including F, Cl, Br, and I)-doped monolayer PtSe_2_ have been calculated by DFT [36]. As compared with pristine monolayer PtSe_2_, the localized impurity states located close to the CBM are identified. The band structure of transition metal-doped PtSe_2_ has been simulated by Kar et al. by using DFT [69]. And they found that group IIIB, VB, VIII8, VIII9, and IB transition metal-doped monolayer PtSe_2_ exhibits half-metallic properties together with spin gap. Besides, the other transition metal-doped PtSe_2_ exhibits tunable semiconducting or tunable magnetic semiconducting properties.

### Phase Transition

Due to the strong covalent bond strength and weak interlayer interaction, the structure of 2D materials strongly depends on varying external effects (pressure, strain, irradiation, annealing, or lithiation) [23]. Phase transition can be induced by ionic intercalation, high pressure, strain, thermal treatment, and external electrical and magnetic field. Since 1T-PtSe_2_ is a very stable structure, it is difficult to expect a continuous phase transition unless inducing additional electron beam irradiation and annealing treatment.

**Fig. 4:**
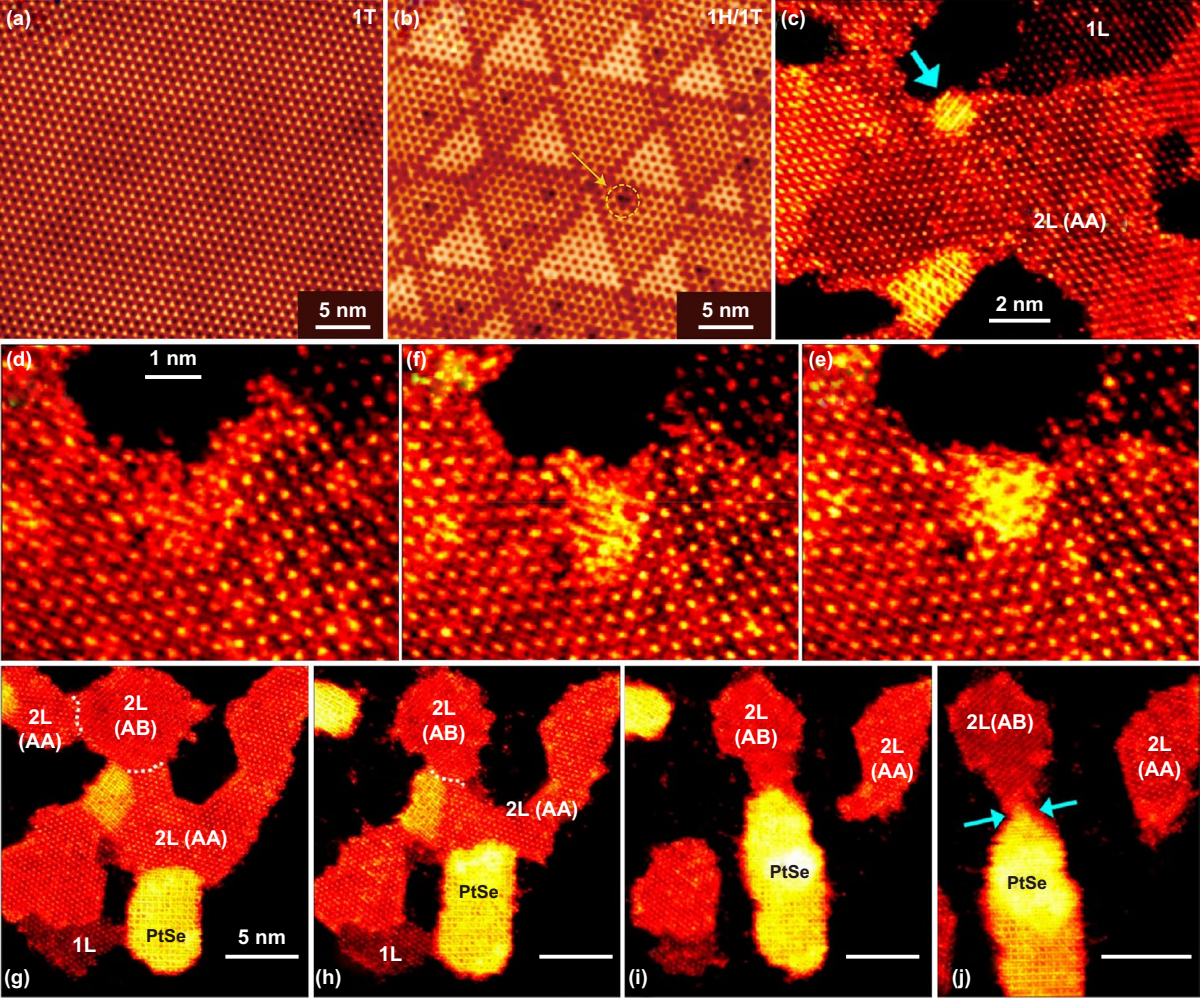
In situ characterization of phase transitions of PtSe_2_. STM images of **a** 1T-PtSe_2_ and **b** 1H/1T patterned PtSe_2_. Reproduced with permission [70]. Copyright 2017, Springer Nature. **c** PtSe_2_ at the edge of bilayer PtSe_2_. **d, e** Successive annular dark field STEM images at a position indicated by a cyan arrow in (**c**). **f** Time between images ~ 60 s. **g–j** Successive annular dark field STEM images at the position indicated by cyan arrows in (**j**). White dashed line indicated the grain boundary. **c–j** Reproduced with permission [75]. Copyright 2019, American Chemical Society

As show in Fig. 4a, b, Lin et al. studied the reversible phase transition of 1T phase and 1T/1H patterned PtSe_2_ by using in situ STM [70]. The homogeneous 1T-PtSe_2_ shown in Fig. 4a was directly synthesized on a Pt (111) substrate via a TAC process at 270 °C. As the 1T-PtSe_2_ film annealed at 400 °C, periodic triangular pattern structure of alternating 1H/1T patterned phases formed. The STM images of the 1H/1T patterned phases are shown in Fig. 4b. Moreover, the triangular 1H/1T pattern reverts to a homogeneous 1T phase PtSe_2_ by annealing the periodic triangular 1H/1T patterned PtSe_2_ at 270 °C in Se steam atmosphere. However, Lin et al. found that the 1H/1T triangular pattern can be directly prepared by controlling the initial density of Se atoms during the TAC process. The DFT and experiment measurement show that the Se vacancies mediate the formation of 1H domains. This transformation process has been reported in other 2D materials, such as monolayer MoS_2_ [71, 72] and PdSe_2_ [73, 74].

In addition, Ryu et al. [75] demonstrated that 1T phase PtSe_2_ can transform into non-layered 2D PtSe_2_ ultrathin film. The phase transformation from 1T PtSe_2_ into non-layered PtSe_2_ crystals induced due to the Se loss during the additional heating process at high temperature (550 °C). As shown in Fig. 4c, d, the rearrangement and restacking of the atoms have been in situ observed by taken successive annular dark field scanning transmission electron microscope (ADF-STEM) images. It can be found that the phase transition occurred only in the bilayer region. Further characterization of the phase transition process has been observed by constructed AA stacking and AB stacking bilayer PtSe_2_. As shown in Fig. 4e–g, the phase transition occurs only in the AA stacking PtSe_2_ region. As the PtSe_2_ film was heated, the non-layered PtSe_2_ structure continued to expand and blocked at the grain boundary.

Besides the annealing and heating process, plasma treatment process has also been proved as an efficient method to induced phase transition in 2D PtSe_2_. Yang et al. [76] reported an inductively coupled plasma treatment method to selectively controlling the thickness of PtSe_2_ flakes. With the decrease in thickness, PtSe_2_ flake transforms from a semimetal to semiconductor. This is well consistence with the prediction concerning their intrinsic thickness-dependent band structure. However, Shawkat et al. discovered a reversed transition of semiconducting to metallic as the PtSe_2_ film irradiated by plasma. Shawkat et al. [77] realized a semiconductor-to-metallic transition in wafer-scale PtSe_2_ film by controlled plasma irradiation. Extensive structural and chemical characterization has proven that large concentration of near atomic defects and selenium vacancies introduced by the plasma irradiations induced the transition of semiconductor to metallic.

The phase transition driven by thermal heating makes it possible for fabricating lateral heterojunctions composed of 1T-PtSe_2_, 1H-PtSe_2_, and PtSe. The electronic properties of 2D PtSe_2_ materials can be modulated by the induced phase transition, which offers new opportunities in both fundamental research and (opto-) electronic devices applications.

### Vibration Spectroscopic Modes

Raman spectroscopy is a powerful and nondestructive optical characterization technique to study the lattice vibrations as well as electron–phonon coupling of 2D materials. Due to the strong interlayer coupling and hybridization, the Raman spectra of PtSe_2_ exhibit interesting anomalous changes.

**Fig. 5:**
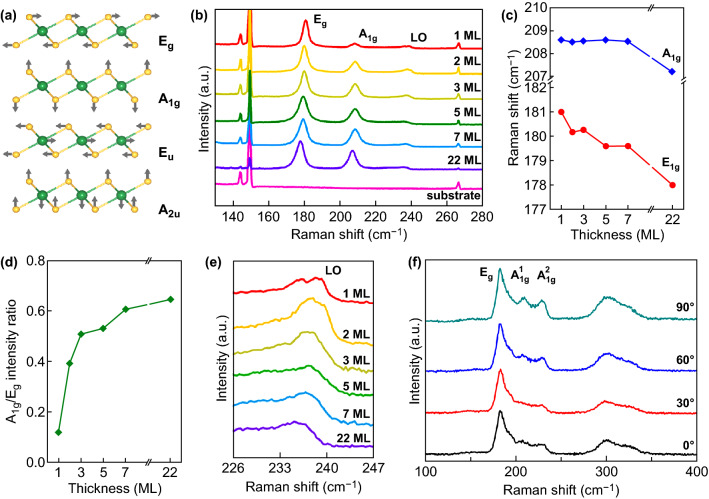
Vibrational properties of PtSe_2_. **a** Schematic views of Raman vibrational modes in PtSe_2_. **b** Thickness-dependent Raman spectra of PtSe_2_. **c** The *E*_*g*_ and *A*_*1g*_ peak positions of PtSe_2_ with increase in thickness. **d** Analysis of thickness dependent of *A*_*1g*_*/E*_*g*_ intensity ratio. **a–d** Reproduced with permission [43]. Copyright 2017, IOP Publishing. **e** Zoom in Raman spectra of the LO mode. **f** Polarization-dependent Raman spectra of PtSe_2_. Reproduced with permission [28]. Copyright 2018, John Wiley and Sons

The schematic diagram of four Raman active vibrational modes in PtSe_2_ is shown in Fig. 5a [43]. The *A*_*1g*_ mode and *E*_*g*_ mode are originated from the out-of-plane vibration and the in-plane vibration of Se atoms, respectively. Figure 5b exhibits the Raman spectra of PtSe_2_ with different thickness. As shown in Fig. 5b, the Raman spectra of 2D PtSe_2_ with different thickness, laser wavelength, and laser polarization were systematically studied. Three primary Raman peaks which allocated to *E*_*g*_ (~ 180 cm^−1^), *A*_*1g*_ (208.5 cm^−1^), and *LO* (~ 240 cm^−1^) modes are observed. As the Raman spectra are normalized to the *E*_*g*_ peak, the relative intensity of the *A*_*1g*_ peak exhibits an obvious decreasing as the thickness decreased. As shown in Fig. 5c, the peak positions of the *E*_*g*_ and *A*_*1g*_ mode are extracted and plotted as a function of number of layers. The position of *E*_*g*_ mode exhibits a clear red shift with increase in thickness. However, the position of *A*_*1g*_ mode is almost unchanged for few-layer PtSe_2_ and exhibits an obvious red shift as the number of layers larger than 22 layers. The layer dependence of Raman spectra properties may be attributed to the strong long-range interlayer interactions [78, 79]. To further study the relationship between the thickness and Raman spectra, the intensity ratio of the *A*_*1g*_ peak to *E*_*g*_ peak is extracted and plotted in Fig. 5d. The extracted thickness\intensity ratio is well consistent with the enhanced van der Waals interactions between the layers in thicker 2D materials [68, 78, 80]. Besides the distinct *E*_*g*_ and *A*_*1g*_ modes, Raman peaks ascribed to less prominent *LO* mode, which attributed to a combination of the in-plane *E*_*u*_ and out-of-plane *A*_*2u*_ vibrations, are also observed [43, 55]. The enlarge spectra in Fig. 5e show the thickness-dependent position of the *LO* peaks. With increase in number of layers, the *LO* peaks change into a broader hump and the intensity decreased.

Moreover, the vibration modes of PtSe_2_ were further characterized by polarization-dependent Raman spectra [28, 78]. As shown in Fig. 5f, the variation of the polarization of the incident light has no effects on the intensity of *E*_*g*_ peak (around 180 cm^−1^), which confirming the in-plane nature of this mode. However, an obvious intensity decrease in the *A*_*1g*_ peak (around 208 cm^−1^) and *LO* peak (around 240 cm^−1^) depending on light polarization is observed. The decrease in the intensity of *A*_*1g*_ peak and *LO* peak confirms the out-of-plane vibration nature of these two modes. It has been proved that these Raman peaks have been observed in the mechanic exfoliated PtSe_2_ single crystal as well as the PtSe_2_ film grown via TAC process [28, 78].

### Optical Properties

#### Layer-Dependent Optical Absorption Spectra

The refractive index and extinction coefficient are fundamental properties of a material that not only determines its optical responses, but also directly connects to its complex permittivity and dielectric constant. Wang et al. measured the refractive index and extinction coefficient of the PtSe_2_ (~ 3 nm) in the wavelength range from 200 to 900 nm by using spectroscopic ellipsometry [31]. The refractive index of the ultrathin PtSe_2_ film increased from 1.5 to 4.5 as the wavelength increased from 200 to 900 nm. However, the extinction coefficient of the ultrathin PtSe_2_ film is almost unchanged and maintained around 2.4. Xie et al. [81] also measured the refractive index and extinction coefficient of the PtSe_2_ film in the wavelength range from 360 to 1700 nm. The refractive index and extinction coefficient values are strongly dependent on the thickness of PtSe_2_ film. By analyzing the spectroscopic ellipsometry results, the values of refractive index and extinction coefficient increased as the film thickness increasing.

**Fig. 6:**
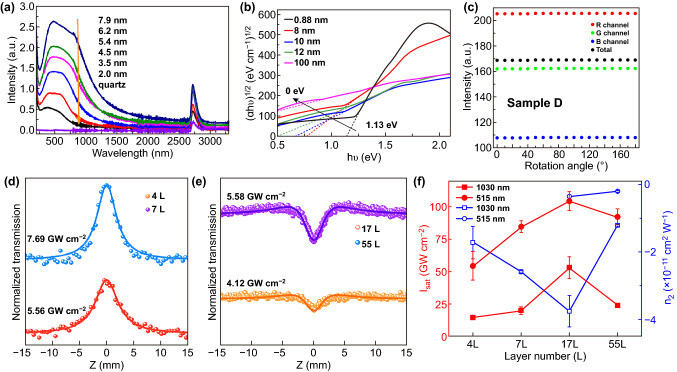
Optical properties of PtSe_2_. **a** Thickness-dependent UV–Vis–IR absorption spectra of PtSe_2_ film on sapphire substrate. Reproduced with permission [82]. Copyright 2019, AIP Publishing. **b** Thickness-dependent Tauc plots of PtSe_2_ film. Reproduced with permission [28]. Copyright 2018, John Wiley and Sons. **c** Intensity of red channel, green channel, blue channel, and the total intensity at different rotation angle. Reproduced with permission [81]. Copyright 2019, IOP Publishing. **d**, **e** Open aperture Z-scan results of 4L, 7L, 17L, and 55L PtSe_2_ films at 1030 femtosecond pulse excitation. **f** Saturation irradiance (*IS*_*at*_) and **g** nonlinear refractive index (*n*_*2*_) of 4L, 7L, 17L, and 55 L PtSe_2_ films at 1030 nm and 515 nm. Reproduced with permission [84]. Copyright 2019, John Wiley and Sons

As shown in Fig. 6, the thickness-dependent optical absorption spectra of PtSe_2_ films were measured in the wavelength range of 200–3300 nm [82]. As shown in Fig. 6a, PtSe_2_ exhibits a broadband absorption response with a smooth absorption band in the wavelength range of 400–800 nm. However, in the wavelength range of 800–2200 nm, the absorption intensity decreased as the wavelength increased. In the range of 2200–3300 nm, PtSe_2_ still exhibits a broadband absorption and the intensity almost unchanged in this wavelength range. Besides, the absorption spectra of thicker PtSe_2_ film exhibit an obvious red shift. Usually, semiconductors cannot absorb light with energy much smaller than the band gap, but PtSe_2_ film exhibits strong light absorption in a broadband wavelength range from deep ultraviolet to mid-infrared (mid-IR) [28, 66, 81–83]. The strong IR light absorption of PtSe_2_ mainly attributes to the semimetallic components of the films [81, 82].

The band gap of semiconductors can be easily experimentally measured by using optical absorption spectra. The layer-dependent Tauc plots of PtSe_2_ are presented in Fig. 6b. With increase in thickness, the absorption edge of Tauc plot shows an obvious red shift. The band gaps of monolayer PtSe_2_ are well consistence with the DFT calculation results. Meanwhile, the transition from semiconductor to semimetal of PtSe_2_ has been verified by the layer-dependent Tauc plots, which is also well agreement with DFT calculation.

#### Isotropic Optical Properties

Xie et al. [81] studied the optical isotropy properties by using polarized optical imaging method and polarization-dependent optical absorption measurement, and they ascertained the optical isotropy in the 2D PtSe_2_. As shown in Fig. 6c, the intensity of the red, green, blue (RGB) channels and the total intensity at different rotation angles were extracted from the polarized optical images of a PtSe_2_ film (~ 5.3 nm). As the rotation angle changed, the intensity of RGB channels and total intensity are almost unchanged, which indicate the optical isotropy of PtSe_2_. The absorption spectra in the range of 400–800 nm under polarization directions of 0° (horizontally), 90° (vertically), and non-polarized light for PtSe_2_ film (~ 5.3 nm) were measured. These absorption spectra are well consistent with each other, indicating the in-plane isotropic optical absorption in PtSe_2_ film.

#### Nonlinear Optical Properties

Nonlinear optical (NLO) properties of 2D materials have been taken as the forefront of the research, which are crucial for developing high-performance ultrafast laser and optoelectronic devices [85–93]. PtSe_2_ has nonlinear effects in a wide wavelength range due to its narrow band gap. Tao et al. [94] investigated the NLO properties of TAC-synthesized PtSe_2_ films. A modulation depth of 12.6% and saturation fluence of 17.1 μJ cm^−2^ were obtained based on the NLO transmittance curve. The saturable absorption (SA) characteristics of the transverse-electric and transverse-magnetic modes of PtSe_2_ are studied by Zhang et al. [95] Modulation depth of 4.90% (transverse-electric modes) and 1.11% (transverse-magnetic modes) are obtained based on the NLO transmittance curves.

The NLO properties of few-layer PtSe_2_ have been systematic studied by using the Z-scan method and pump–probe–technique [84]. As shown in Fig. 6d, two small peaks near the symmetrical valley are observed in the open aperture (OA) signals of 4L and 7L PtSe_2_ films, which suggest the OA signals consist of both SA and two-photon absorption (2PA) response at 1030 nm. However, there are no peaks observed near the symmetrical valley in the OA signals of 17L and 55L PtSe_2_ (Fig. 6e), which indicate the pure SA response. The evolution of the saturation (IS_at_) and irradiance nonlinear refractive index (*n*_2_) are extracted and plotted in Fig. 6f, g. The large IS_at_ at 515 nm indicates that PtSe_2_-based saturable absorber possesses higher saturation intensity in visible range than that in near-IR range. Besides, the large value *n*_2_ of PtSe_2_ suggests the huge potential to developing self-defocusing materials in NLO devices.

## Synthesis Methods

Generally, the crystal structure, crystallinity, and properties of 2D PtSe_2_ are strongly related to the synthesis process. It is still challenging to prepare 2D PtSe_2_ with the desirable thickness, lateral size, and microstructure for specific applications. Indeed, various strategies have been proposed to prepare 2D PtSe_2_, specially aiming to materials with high quality and large lateral size. Up to now, great achievements have been made to prepare 2D PtSe_2_ with controllable thickness, morphology, and lateral size. In this section, different synthesis methods for preparing 2D PtSe_2_ are systematically discussed. CVD and TAC are the most widely studied methods to synthesis 2D PtSe_2_. Moreover, the other methods such as mechanical exfoliation and CVT have also been studied.

### Mechanical Exfoliation

**Fig. 7:**
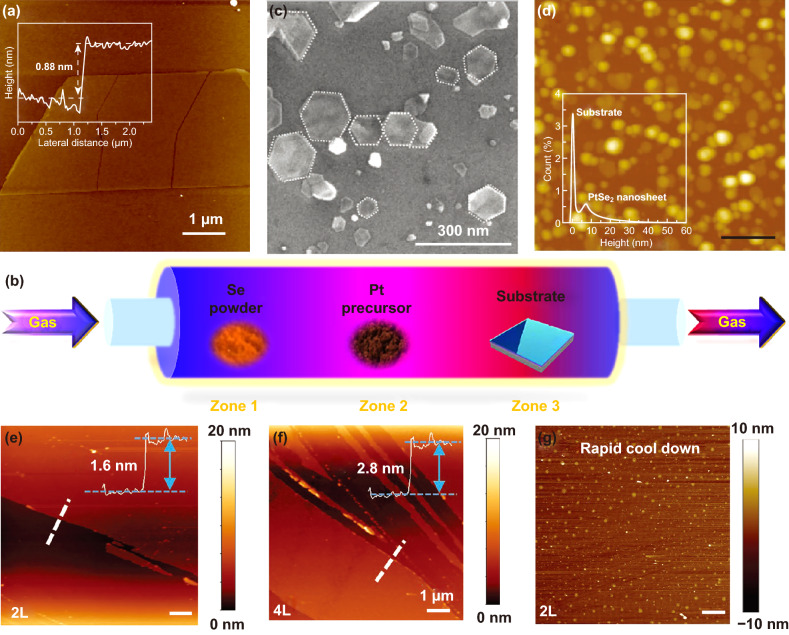
2D PtSe_2_ prepared by Mechanical exfoliation/CVD. **a** AFM image of the exfoliated monolayer PtSe_2_ and corresponding height profiles. Reproduced with permission [28]. Copyright 2018, John Wiley and Sons. **b** Schematic diagram of the three zones CVD system for the synthesis of 2D PtSe_2_. **c** SEM morphology of the PtSe_2_ nanosheets synthesized by CVD process. **d** AFM image of the PtSe_2_ nanosheets synthesized by CVD process. **c**, **d** Reproduced with permission [106]. Copyright 2016, John Wiley and Sons. The AFM image of the **e** bilayer and **f** four layer PtSe_2_ film and corresponding height profiles [107]. **g** AFM image of the bilayer PtSe_2_ synthesized via rapid cool down process. **e–g** Reproduced with permission [107]. Copyright 2019, John Wiley and Sons

Mechanical exfoliation (ME) is one of the most commonly used methods to prepare high quality 2D materials [96–99]. Monolayer or few-layer 2D materials obtained by this method can maintain their intrinsic structure and are suitable for fundamental research. Mechanical exfoliation has been extensively exploited to prepare monolayer or few-layer 2D materials, such as graphene, BP, nitride, TMDCs, and MXene. The mechanical exfoliation process is a relatively simple and fast process by repeating adhesion and splitting. As the monolayer or few-layer 2D materials attached on the surface of scotch tape, the as-prepared 2D materials can be easily transferred to selected substrate [1, 99]. In 2017, Zhao et al. [28] prepared monolayer PtSe_2_ by using mechanical exfoliation. The high quality PtSe_2_ bulk crystal was grown by CVT method. Ultrathin PtSe_2_ was peeled from bulk PtSe_2_ using a scotch tape, as shown in Fig. 7a. Huang et al. [100] prepared PtSe_2_ nanoflakes with the thickness of ~ 70 nm by mechanical exfoliation its single crystal. However, mechanical exfoliated 2D materials can only fulfill the using demands of fundamental studies due to the limited yield and relatively small lateral size.

### Chemical Vapor Deposition

CVD is an important synthesis method to prepare high quality 2D materials with scalable size, controllable thickness, and perfect crystal structure for both fundamental research and practical applications [101–104]. To date, various materials with controllable layer number, lateral size, and microstructure have been successfully prepared via CVD methods, such as graphene, TMDC, Xene, boron nitride, and MXene. Recently, the CVD growth of monolayer or few-layer 2D PtSe_2_ has also attracted extensively attention and has been taken as a promising method to realize the large-scale growth of 2D PtSe_2_.

PtSe_2_ with controlled morphology can be synthesized by CVD process via precise tuning of the growth temperature, pressure, and precursors [83, 105–108]. Figure 7b shows the typical schematic illustration of a 3-zone CVD growth setup, wherein the precursors are placed in different zone of the quartz tube. Typically, Se powder and PtCl_4_ or H_2_PtCl_6_ powder are chosen as the precursors; the obtained PtSe_2_ is found to be nearly hexagonal with the thickness ranging from 3.5 to 10 nm [106]. However, by tuning the growth temperature of zone 3 from 900 to 500 °C, Xu et al. successfully prepared polycrystalline PtSe_2_ film with controlled thickness by tuning the growth time [107]. The morphology of the single-crystalline and polycrystalline PtSe_2_ is shown in Fig. 7c–g, respectively. As shown in Fig. 7e–g, large area continuous PtSe_2_ films with controlled thickness have been successfully synthesized via a one-step CVD process. Furthermore, the cooling down rate also has great effect to the surface morphology. The rapid cooling rate may suppress the diffusion of reactive atoms, leading to the formation of the multilayer island on the surface [107]. The multilayer islands on the surface of PtSe_2_ thin film are shown in Fig. 7g.

The morphology, thickness, microstructure, and lateral size of 2D materials can be well controlled by precise controlling CVD growth parameters. The quality of the as-grown PtSe_2_ can be determined by many factors including but not limited to the precursors, pressure, temperature, heating rate, and substrate. Thus, in-depth understanding of the CVD growth mechanism is beneficial to the improvement of scalability and controllability for PtSe_2_ synthesis.

### Thermally Assisted Conversion

TAC of pre-deposited metal on substrate is also an effective strategy to grow wafer-scale 2D materials [109–111]. PtSe_2_ prepared by this method is a just simple chemical reaction, Pt + 2Se = PtSe_2_. Direct selenization of the Pt film provides a simple and fast approach to obtain wafer-scale 2D PtSe_2_ film.

**Fig. 8:**
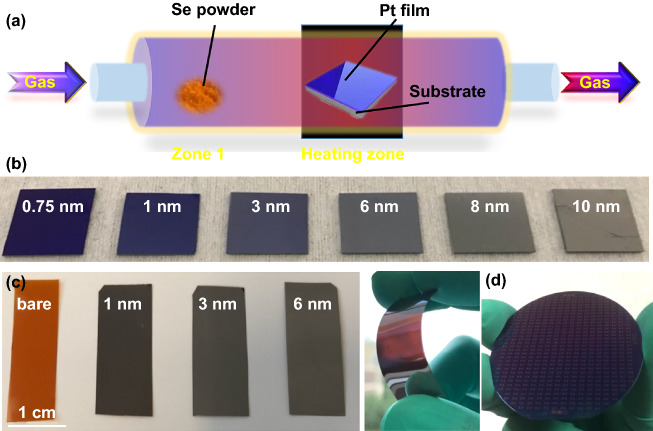
Large-scale PtSe_2_ film growth by TAC. **a** Schematic diagram of the CVD system for the synthesis of 2D PtSe_2_ film by using TAC process. **b** Photograph of 2D PtSe_2_ film on SiO_2_/Si substrate with controlled thickness. **c** Photograph of 2D PtSe_2_ film on polyimide substrate with controlled thickness. Reproduced with permission [114]. Copyright 2019, American Chemical Society. **d** Photograph of a wafer-scale PtS_2_/PtSe_2_ film on a 2-in. SiO_2_/Si substrate. Reproduced with permission [117]. Copyright 2018, American Chemical Society

The TAC process is a straightforward and simple route for synthesizing large-scale PtSe_2_ with controlled thickness. Pt film with different thickness is initially deposited on a given substrate via a magnetron sputtering process or electron beam evaporate process. Then, the PtSe_2_ film is prepared via the directly selenization process. As shown in Fig. 8a, the Se powder is placed at the upstream side in the tube furnace, and the Pt coated substrates are placed in the heating zone. During the selenization process, the growth temperature is usually set to about 270-500 °C, while the pressure remains at about 80 mTorr with argon gas protection [25, 112, 113]. In 2015, Wang et al. [25] firstly fabricated a single crystal monolayer PtSe_2_ by direct selenization of Pt (111). Han et al. [114] prepared large-scale 2D PtSe_2_ with different thickness on SiO_2_/Si substrate. Figure 8b shows the photograph of the PtSe_2_ film with different thickness on SiO_2_/Si substrate. The lateral size and thickness of PtSe_2_ film can be controlled by modulating thickness of the pre-deposited Pt film [84, 115].

Since the pre-deposition process and post-selenization process are carried out in relatively mild condition, the PtSe_2_ film can be prepared on arbitrary substrates. Besides the conventional Si [33, 115, 116], Si/SiO_2_ [32, 33, 55, 94, 112, 114, 117–124], and Sapphire substrate [81, 125, 126], 2D PtSe_2_ film has been successfully grown on fused quartz [31, 84, 125], fluorine-doped tin oxide (FTO) [127, 128], gallium nitride (GaN) [129], and polyimide [114]. Figure 8c shows the PtSe_2_ on the surface of flexible polyimide [41, 114]. As shown in Fig. 8d, Yuan et al. [117] fabricated a wafer-scale PtSe_2_/PtS_2_ heterojunction film via two step TAC process on a SiO_2_/Si wafer with 300-nm-thick Si dioxide. TAC process enables the growth of PtSe_2_ on wafer-scale substrate, offering the throughput that can meet the demand for practical application.

### Other Methods (Molecular Beam Epitaxial, CVT)

**Fig. 9:**
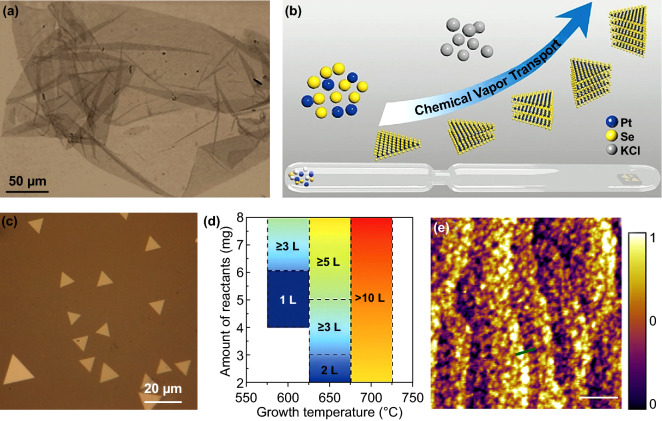
2D PtSe_2_ prepared by solution process/CVT/MBE. **a** Optical image of the PtSe_2_ nanosheets prepared via an aqueous-phase reaction process. Reproduced with permission [135]. Copyright 2017, American Chemical Society. **b** Schematic diagram of the controlled synthesis of PtSe_2_ by CVT. **c** Optical image of PtSe_2_ triangle nanoflakes prepared by CVT. **d** Relationship of the layer numbers of the as-grown PtSe_2_ triangle nanoflakes as a function of growth temperature and amount of reactants. Reproduced with permission [142]. Copyright 2019, John Wiley and Sons. **e** AFM image of the PtSe_2_ grown on the bilayer graphene by MBE method. Reproduced with permission [43]. Copyright 2017, IOP Publishing

In addition to the aforementioned methods, some other approaches also have been reported to synthesize 2D PtSe_2_. For example, some pioneer works have been reported that PtSe_2_ can be prepared via a Sol–Gel solution process [130–134]. Umar et al. [135] reported the successful synthesis of scalable 2D PtSe_2_ nanosheets via an aqueous-phase synthetic strategy for the first time. PtSe complexes precursors are initially prepared via surfactant-template self-assembly process. Then, the mesoporous 2D PtSe_2_ nanosheets are prepared by thermal annealing the PtSe complexes precursors. As shown in Fig. 9a, the 2D PtSe_2_ nanosheets with a thickness about 11–25 nm are synthesized, indicating that scalable PtSe_2_ can be produced by a straightforward process to scalable produce PtSe_2_. Pawar et al. [136] also prepared 2D PtSe_2_ nanosheets by using the almost same method that Umar reported.

As a widely studied traditional crystal growth method, CVT has also been employed to direct synthesize 2D semiconductor materials, such as TiSe_2_, MoS_2_, WS_2_, and ReS_2_ [137–141]. Benefitting from the good controllability of the growth parameters, the properties, structure, and composition of 2D materials can be well regulated. In 2016, Yu et al. [33] successfully synthesized single crystal of PtSe_2_ by using CVT method. This achievement makes it possible for us to grown 2D PtSe_2_ by precise controlling the growth condition. Hu et al. [142] successfully synthesizes 2D PtSe_2_ nanosheets with controlled thickness by using CVT. As shown in Fig. 9b, the schematic diagram of the CVT process is presented. The raw materials are put in a sealed the quart tube, while the substrate is placed in the other side of the quart tube. By carefully adjusting the amount of precursors and transport agent, triangular-shaped single-crystalline PtSe_2_ flakes were obtained on the mica substrate. The optical morphology of the triangular-shaped single-crystalline PtSe_2_ flakes is shown in Fig. 9c, and the relationship of the thickness with temperature and reactants is exhibited in Fig. 9d. However, only few papers have reported the synthesis of 2D PtSe_2_ by using CVT due to the complex growth condition. Since growth of bulk semiconductor crystal by CVT is much easier than direct growth 2D semiconductor materials, CVT is generally employed to grow high quality single-crystalline bulk materials, ultrathin 2D flakes are then peeled from bulk crystal by mechanical exfoliation [142–144]. For example, Zhao et al. [28] grow PtSe_2_ single crystal by using CVT method and the air stable 2D PtSe_2_ are peeled from the bulk PtSe_2_ crystal.

Molecular beam epitaxy (MBE) has been playing an important role in the growth of high quality 2D materials film with controlled thickness [145, 146]. Yan et al. [43] successfully prepared high quality PtSe_2_ films on bilayer graphene/6H(0001) substrate by using MBE method for the first time. The surface morphology of the as-prepared PtSe_2_ on the surface of bilayer graphene is shown in Fig. 9e. The obtained PtSe_2_ film is single crystalline and the thickness ranges from 1 to 22 layers.

The reliable production of 2D PtSe_2_ with controlled structure is a prerequisite in exploring their properties and possible applications. As mentioned above, 2D PtSe_2_ has been prepared by various approaches including mechanical exfoliation, CVD, CVT, TAC, and other methods. A comprehensive summary and comparison with these methods is presented in Table 1. The aforementioned methods have inherent disadvantages which make it difficult to achieve the large area and highly crystalline structure. And the synthesis of large lateral size and uniform monolayer or few-layer 2D PtSe_2_ is still challenge. Moreover, the growth mechanism has yet to be clarified. Therefore, extended works need to be done to achieve the controllable synthesis of 2D PtSe_2_.

**Table 1:** Comparison of different synthesis methods to prepare 2D PtSe_2_

Methods	Lateral size	Number of layers	Achievements	Challenges
ME	~ Micrometers	Any number of layer	High quality	Uncontrollable
CVD	~ Micrometers	1L ~ few layer	Single crystal high quality	Limited area
TAC	Wafer scale	Few layer to tens of nanometers	Continuous film of wafer-scale size	Polycrystalline film
				Surface roughness
CVT	~ Centimeter	Nanoflakes to bulk	Large crystal	Long growth time
			High quality	Difficult to growth few-layer samples
MBE	~ Centimeter	Few layer	Large scale	Complex
			High quality	Expensive
			Controlled thickness	Limited substrate

## Applications

### Photodetectors

Photodetectors can directly convert optical signals to electrical signals. It has been widely applied in many fields such as optical communication, industrial automatic control, and military [147–149]. 2D materials, including graphene, BP, and TMDCs, are considered to be promising candidates for high-performance photodetectors due to their excellent properties and complementary metal oxide semiconductor compatible [147, 150–155]. However, it is still challenge to fabricate high responsivity 2D material-based photodetectors along with ultrafast response. Although group-6 TMDCs (such as MoS_2_ and WS_2_) have exhibited impressive optoelectronic properties [156–158], their photodetection performance is severely limited due to their relatively large band gap and low carrier mobility, especially in the IR range.

As newly emerged 2D materials, group-10 TMDCs have been widely studied as high-performance photodetectors [29, 117, 121, 159]. Among these group-10 TMDCs materials, PtSe_2_ has been demonstrated to have excellent photoelectric and electrical properties. As introduced above, the band gap of monolayer and bilayer PtSe_2_ is 1.2 and 0.21 eV, respectively [25]. Simulation results have revealed that only monolayer PtSe_2_ has a sizeable band gap and PtSe_2_ become semimetallic as the number of layers larger than three or four. Thus, 2D PtSe_2_ is proposed as an excellent candidate for broadband photodetectors in the visible to mid-IR range [32, 33, 116–118, 120, 121, 123, 129, 160]. As shown in Table 2, the performance of PtSe_2_-based photodetectors is summarized for comparison.

**Table 2:** Summary of PtSe_2_-based photodetectors

Materials	Wavelength (nm)	Photoresponsivity (mA W^−1^)	Rise/fall times (μs)	References
PtSe_2_/CdTe	200–2000	506.5@780 nm	8.1/43.6	[121]
PtSe_2_/Silicon	200–1550	12,650@780 nm	10.1/19.5	[160]
PtSe_2_/GaAs	200–1200	262@808 nm	5.5/6.5	[120]
PtSe_2_/Ge	1300–2200	602@1550 nm	7.4/16.7	[118]
PtSe_2_/Perovskite	300–1200	117.7@808 nm	78/60	[123]
PtSe_2_	360–2000	490@970 nm	–	[32]
PtSe_2_/GaN	200–800	193@265 nm	45/402	[129]
PtSe_2_/Si	200–1550	520@808 nm	55.3/170.5	[116]
PtSe_2_	632-10^4^	6250@632 nm	1.1/1.2 × 10^3^	[33]

**Fig. 10:**
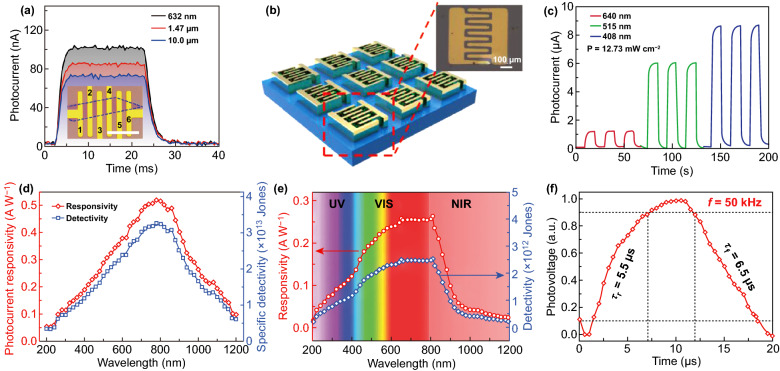
Performance of PtSe_2_-based photodetectors. **a** Time resolved photoresponse of the bilayer PtSe_2_-based photodetectors; the inset is the microscopic image the device. Reproduced with permission [33]. Copyright 2018, Springer Nature. **b** Schematic structure and an optical microscopic image of the 2D PtSe_2_ film-based photodetector. **c** Time resolved photocurrent of the photodetector as a response to light on/off at wavelengths of 408 nm, 515 nm, and 640 nm. **b**, **c** Reproduced with permission [55]. Copyright 2018, John Wiley and Sons. **d** Photocurrent responsivity and specific detectivity of the PtSe_2_ film-based photodetector as functions of the wave length of the incident light. Reproduced with permission [116]. Copyright 2018, Royal Society of Chemistry. **e** Responsivity and detectivity of the PtSe_2_/GaAs heterojunction as a function of wavelength. **f** A single normalized cycle measured at 50 kHz for estimating both response time (*τ*_*r*_) and recovery time (*τ*_*f*_). **e–f** Reproduced with permission [121]. Copyright 2018, John Wiley and Sons

Yu et al. investigated the photoresponse of FETs based on bilayer PtSe_2_ in the wavelength range from 632 nm to 10 μm, as shown in Fig. 10a [33]. The photoresponsivity of 6.25 A W^−1^ and a rise time of about 1.2 ± 0.1 ms were achieved for 640 nm laser illumination. Moreover, the photoresponsivity in the near-IR (~ 1.47 μm) wavelength range and mid-IR (~ 10 μm) wavelength range is about 5.5 and 4.5 A W^−1^, respectively. The fitted rise and fall time for the bilayer PtSe_2_-based photodetector are much better than those 2D materials (such as BP, MoS_2_, and MoSe_2_)-based photodetectors [15, 147, 149, 150, 161–166]. These results indicate that 2D PtSe_2_ is highly promising platforms for high sensitive and broadband optoelectronic application in the range of visible light to mid-IR wavelengths.

Su et al. investigated the performance of PtSe_2_ film-based photodetector on SiO_2_/Si [55]. The schematic structure of the photodetector device and the corresponding optical image are shown in Fig. 10b. As shown in Fig. 10c, the broadband photoresponse is demonstrated in the wavelengths range from 408 to 640 nm. When the photodetector was irradiated by 408 nm laser, the device exhibited the highest photoresponse with the photocurrent reaches 9 μA, while the photocurrent was about ~ 6 and ~ 1 μA as irradiated by 640 and 510 nm laser, respectively. The corresponding photoresponsivity with incident power density of 12.73 mW cm^−2^ is 0.1A W^−1^ (at 640 nm), 0.25 A W^−1^ (at 515 nm), and 0.4 A W^−1^ (at 408 nm). Moreover, the PtSe_2_ can be directly grown on a flexible polyimide substrate owing to the advantage of the low-temperature growth process. Su et al. [55] also fabricated a flexible photodetector based on PtSe_2_ film on the polyimide substrate by using the same conditions of photodetectors fabricated on the SiO_2_/Si substrate. The photodetector exhibits great stability under different bending radius with almost no degradation in the photocurrent even after 1000 bending cycles.

Yim et al. studied the photoresponse of the layered PtSe_2_-based Schottky barrier diodes on *n*-type Si [32, 112]. The diode was fabricated by transferring PtSe_2_ thin films onto the pre-patterned *n*-type Si substrate. The PtSe_2_ film exhibits strong photoresponse over a broadband wavelength range of 360–2000 nm. The maximum photoresponsivity of 0.49 A W^−1^ and minimum photoresponsivity of 0.0001 A W^−1^ were measured at photon energies above and below the band gap of Si. In the visible region, the large part of the photocurrent in the PtSe_2_/Si device is generated in the Si layer, whereas the photocurrent in IR region is generated in the PtSe_2_ layer [32]. Xie et al. and Zeng et al. in situ fabricated vertical PtSe_2_/Si hybrid heterojunctions [33, 116]. The PtSe_2_ films were grown directly on Si substrates, which can effectively avoid the interface contamination, structural continuity deterioration, and materials surface tear. This heterojunction-based photodetector is highly sensitive in a broad wavelength region from deep ultraviolet (200 nm) to near-IR (1550 nm). As shown in Fig. 10d, the highest photoresponsivity of the PtSe_2_/Si can reach 0.52 A W^−1^ at 808 nm, and the specific detectivity and rise/fall response times are 3.26 × 10^13^ Jones and 55.3/170.5 μs [116]. When Si nanowires were chosen to fabricate PtSe_2_/Si heterojunction, a high photoresponsivity of 12.65 A W^−1^ and very fast rise/fall time of 10.1/19.5 μs are obtained in the PtSe_2_/Si nanowires-related photodetector [33].

The broad band gap range and high carrier mobility of PtSe_2_ make it be an excellent candidate for developing high-performance photodetectors. However, the ultrathin thickness of 2D PtSe_2_ materials result in a low absorption to incident light, leading to a small photocurrent, large dark current and low specific detectivity. In order to develop broadband, high sensitive, low power, and high photoresponsivity photodetector, PtSe_2_-based heterostructure for optoelectronic applications has been studied [117, 118, 120, 121, 129]. Wu et al. [121] designed a vertical PtSe_2_/CdTe heterojunction-based photodetector and this photodetector exhibited a broad detection wavelength range of 200–2000 nm. This heterojunction structure can enhance the absorption to near-IR light, as well as the improvement of response speed due to the formation of a built-in electric field. Zeng et al. [121] fabricated a PtSe_2_/GaAs heterojunction on SiO_2_/Si substrate via a deposition process and wet transfer process. The PtSe_2_/GaAs heterojunction-based photodetector exhibited high sensitivity to broad wavelength range from 200 to 1200 nm. As shown in Fig. 10e, the photodetector exhibits peak sensitivity in the range from 650 to 810 nm, which exclusively originates from the PtSe_2_ layer. The rise/fall time for the photodetector is 5.5/6.5 μs (shown in Fig. 10f), which are faster than other state-of the art 2D materials (such as BP, MoS_2_, WS_2_, and graphene/Si heterojunction) photodetectors [167–170]. Wang et al. [118] fabricated a PtSe_2_/Ge heterojunction-based photodetector, which is highly sensitive to the near-IR light. The photodetector device can operate without an external power supply due to the photovoltaic effect under the near-IR light illumination [112, 118, 120].

### Mode-Locked Laser

The mode locking based on SA has been taken as the most important and efficient optical technique to generate ultrafast pulse laser from a continuous wave laser [171–176]. The mode-locked laser systems have been widely applied in areas including ultrafast pump sources, high-accuracy measurement, ultrafine laser micromachining, and laser surgery [171]. This technique exhibits many advantages such as low cost, high power scalability, high reliability, good mechanical stability, and excellent beam quality. 2D materials with saturable absorber properties have been widely utilized as saturable absorber in the laser cavity for ultrafast pulse generation.

**Fig. 11:**
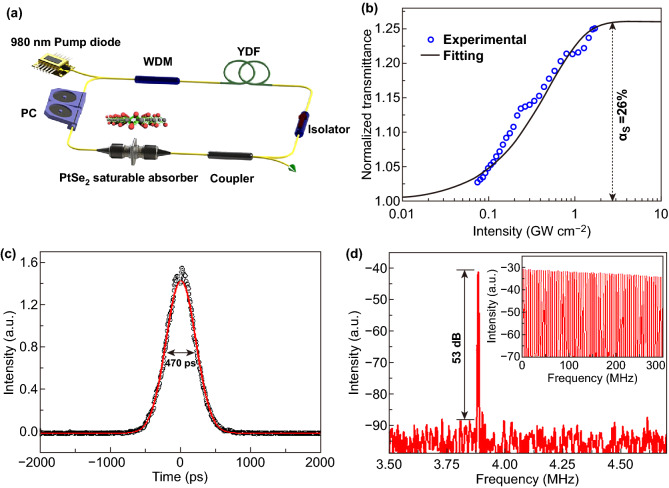
Mode locker laser of PtSe_2_. **a** Schematic diagram of the YDF laser ring cavity. PC: polarization controller. WDM: wavelength division multiplex. Isolator: polarization-independent isolator. YDF: ytterbium-doped fiber. **b** Nonlinear transmission curve of 10 nm PtSe_2_ saturable absorber. **c** Single pulse profile indicates the pulse duration. **d** Radio-frequency spectrum of the mode-locked pulses and inset shows the corresponding wideband (0–300 MHz) radio-frequency spectrum. Reproduced with permission [177]. Copyright 2018, American Chemical Society

In 2018, Yuan et al. [177] reported the SA properties of 2D PtSe_2_ film for the first time. Figure 11a shows the schematic diagram of the experimental setup of the ytterbium-doped fiber (YDF) laser ring cavity. As shown in Fig. 11b, the NLO measurements results show that PtSe_2_ film (about 10 nm) exhibits a large modulation depth up to 26% at the wavelength of 1064 nm with a lower saturable intensity, while the saturable intensity is as low as 0.316 GW cm^−2^. The modulation depth refers to the maximum change of transmission or total amount of light loss by saturable absorption of the absorber. The relatively large modulation depth at the wavelength of 1064 nm indicates the potential of PtSe_2_ to be an excellent nonlinear absorption material. As shown in Fig. 11c, d, the mode-locking performance of PtSe_2_ film is investigated by transferring a PtSe_2_ film onto the fiber tip. The mode-locked pulse centered at 1064.47 nm has the pulse duration of 470 ps.

Tao et al. [94] also reported the properties of the passively mode-locked solid state laser by using a 24-nm-thick PtSe_2_ film as the saturable absorber. A pulse duration of 15.8 ps is obtained in the mode-locked fiber laser based on a PtSe_2_ film coated fiber. Zhang et al. [95] fabricated and studied 1563 nm Er-doped fiber laser based on PtSe_2_ film, with pulse duration of 1.02 ps and maximum single pulse energy of 0.53 nJ. Huang et al. [100] fabricated a femtosecond fiber mode locking by transferring thicker PtSe_2_ (~ 73 nm) onto a D-shaped fiber. Due to the nonlinear modulation from the PtSe_2_, the pulse duration of 861 fs and single-to-noise ratio of 61.1 dB were achieved for the 1567 nm mode-locking laser. The recent progresses on the PtSe_2_-based mode-locking laser make PtSe_2_ a promising 2D material for on-chip integration of GHz laser sources toward higher repetition rates and shorter pulse duration [31, 82, 84, 125].

### Field Effect Transistors

**Fig. 12:**
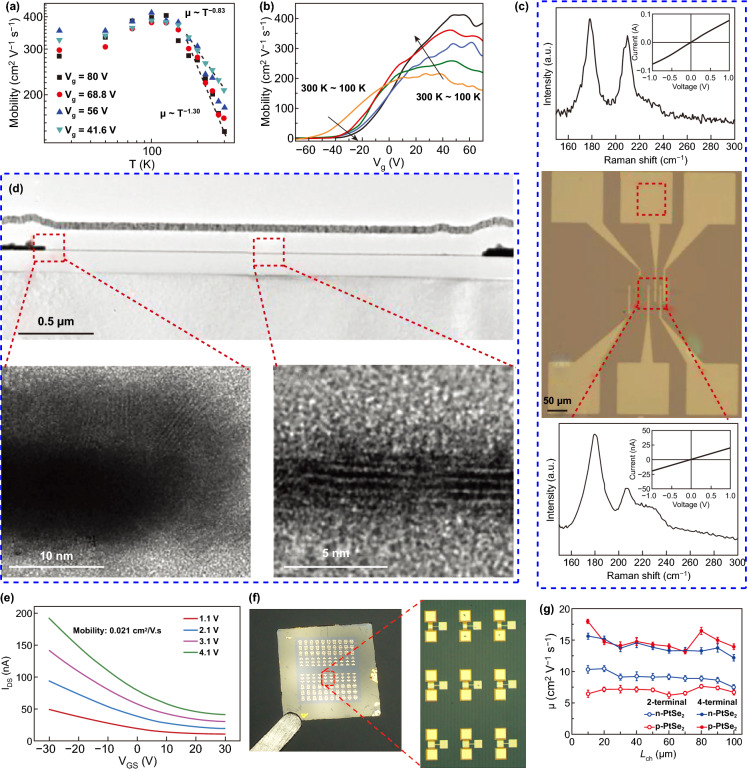
Performance of 2D PtSe_2_ FETs. **a** Temperature-dependent field effect electron mobility of an 11-nm-thick PtSe_2_ FET. **b** Field effect electron mobility versus gate voltage at different the temperature (from 100 to 300 K). Reproduced with permission [28]. Copyright 2018, John Wiley and Sons. **c** An optical microscope image of all PtSe_2_-based devices (middle) and the corresponding Raman spectra of the electrode (upper) and the channel (bottom) regions. **d** Cross-sectional TEM images corresponding to the all PtSe_2_-based device shown in **c**. High magnification TEM images are an enlarge of the electrode (left) and channel (right) regions. **e** *I*_D_*S*–*V*_G_*S* curves at different V_D_S (which are set as 1.1, 2.1, 3.1, and 4.1 V). Reproduced with permission [55]. Copyright 2018, John Wiley and Sons. **f** Optical microscopic images of an PtSe_2_ FETs array. **g** Comparison of the two terminals and four-terminal mobility as a function of the channel length (*L*_ch_) for *p*-type and *n*-type PtSe_2_ film-based devices. Reproduced with permission [107]. Copyright 2019, John Wiley and Sons

One of the important applications of 2D PtSe_2_ materials is the field effect transistors (FETs). The very first report on 2D layered PtSe_2_ material for FETs was reported by Zhao et al. in 2017 [28]. The room temperature electron mobility of the few-layer PtSe_2_ FETs device is 210 cm^2^ V^−1^ s^−1^, which is much smaller than the theoretically predicted value [28, 178]. Zhao et al. further studied the temperature-dependent mobility of PtSe_2_ FETs and the mobility of few-layer PtSe_2_ FETs (~ 11 nm). The field effect mobility and the gate-dependent mobility of the 11 nm-thick-PtSe_2_ FETs are shown in Fig. 12a, b. The mobility of the PtSe_2_ FETs in a back-gated configuration on SiO_2_/Si increased from 210 to 414 cm^2^ V^−1^ s^−1^, as the temperature decreased from 300 to 100 K. Moreover, as the temperature continues to decrease to 25 K, the mobility of the PtSe_2_ FETs decreased from 414 to 353 cm^2^ V^−1^ s^−1^. For comparison, the temperature-dependent mobility of a thinner few-layer PtSe_2_ (~ 8 nm) FETs is measured. When the temperature increased from 25 to 300 K, the mobility increased from 149 cm^2^ V^−1^ s^−1^ (at 25 K) to 233 cm^2^ V^−1^ s^−1^ (at 125 K) and then decreased to 140 cm^2^ V^−1^ s^−1^ (at 300 K). The variation of carrier mobility mainly ascribed to the layer-dependent band gap of PtSe_2_. The reduced band gap of thicker PtSe_2_ leads to the increased carrier density, which improves the screening of charge impurities by the bottom layer. The conductivity and carrier mobility are thus significantly improved due to the carriers in the bottom layer can effectively suppress the Coulomb potential of the charge impurities at the interface [28, 179].

Previous theoretical and experimental results have demonstrated that the thinner PtSe_2_ exhibits a semiconducting behavior, while the thicker PtSe_2_ exhibits a metallic behavior [42, 55]. As shown in Fig. 12c, Su et al. [55] fabricated a full PtSe_2_ FETs wherein the thicker PtSe_2_ (~ 50 nm) is used as the electrodes and the thinner PtSe_2_ (~ 3 nm) is used as the channel materials. To further confirm the existence of the PtSe_2_ in both the channel and electrode, Raman spectra and TEM images of the channel and electrode materials are presented in Fig. 12c, d. The measured electrical properties of the full PtSe_2_ FETs are shown in Fig. 12e. The mobility of the full PtSe_2_ FETs ranges from 0.007 to 0.021 cm^2^ V^−1^ s^−1^, which is lower than the device using pure Pt electrodes [55]. Yim et al. studied the effect of contact metals and edge contact at the metal/PtSe_2_ interface to the transport characteristics of the FETs devices [113]. They found that by increasing the edge contact length, the contact resistivity was improved by up to 70% compared to devices with conventional top contacts, which provide a quick insight into the realization of high-performance opto/electronic devices. Ansari et al. fabricated a back-gated FETs device with different channel thickness [119]. The on/off ratio and carrier mobility are measured at room temperature. The *I*_on_/*I*_off_ ratio of thinner PtSe_2_ film (2.5–3 nm) FETs exceeds 230, while the *I*_on_/*I*_off_ ratio of thicker PtSe_2_ film (5–6.5 nm) FETs is sharply decreased to about 1.4. These variations are mainly due to the quantum confinement effect in the thin 2D PtSe_2_ film. Xu et al. systematically studied the electrical properties of *n*-doping and *p*-doping PtSe_2_ film by fabricating top-gated FET [107]. The optical microscopic image of an as-fabricated FETs array is shown in Fig. 12f. The *I*_on_/*I*_off_ ratio of the PtSe_2_ FETs is about 25 (*n*-type) and 40 (*p*-type). The channel length-dependent electrical properties of the PtSe_2_ FETs have been studied, and the effective field effect mobility of different configurations is presented in Fig. 12g. The four-terminal field effect mobility is nearly three times higher than two terminal field effect mobility for the *p*-type PtSe_2_, and two times higher than the *n*-type PtSe_2_, respectively. Han et al. [114] further identified the interrelation of structural morphology and electrical transport in 2D PtSe_2_ thin film by applying corroborating HR-TEM and FETs characterization. The highest mobility measured in this FETs device reached 625 cm^2^ V^−1^ s^−1^, which is among the highest experimentally measured mobility value reported for PtSe_2_ FETs.

Besides the FETs devices on conventional rigid substrate, Okogbue et al. [180] fabricated a kirigami FETs on flexible polyimide substrate. By taking advantage of the low-temperature synthesis process, they fabricated integrated 2D PtSe_2_ film on flexible. These 2D PtSe_2_/polyimide kirigami patterns exhibit an extremely large stretchability of 2000% without compromising their intrinsic electrical conductance. The corresponding *I*_ds_–*V*_g_ transfer characteristics from the kirigami FETs of varying stretch level (0%, 100%, and 200%) are measured, and these plots clearly reveal that *p*-type semiconducting transports are well retained with slightly decreasing *I*_ds_ during the increasing mechanical stretch.

Recently, impressive advances have been achieved for the fabrication of PtSe_2_ FETs devices. The experimentally measured carrier mobility of PtSe_2_ is much higher than the carrier mobility of group-6 2D TMDC materials, yet it is still much lower than the theoretically predicated value. For 2D materials, there are several extrinsic factors mainly dominating the charge transport, including structurally defects, charge impurity, surface optical phonon scattering, and surface traps [181–184]. These critical issues also existed in 2D PtSe_2_-based device, the negative effects induced by the unexpected impurity (Se dopant), heterojunction interface (electrode/PtSe_2_), and contact resistance still need to be overcome. Besides, due to the low-temperature synthesis process of large-scale 2D PtSe_2_ film, it is would be interesting to develop high-performance flexible devices.

### Photocatalysis

**Fig. 13:**
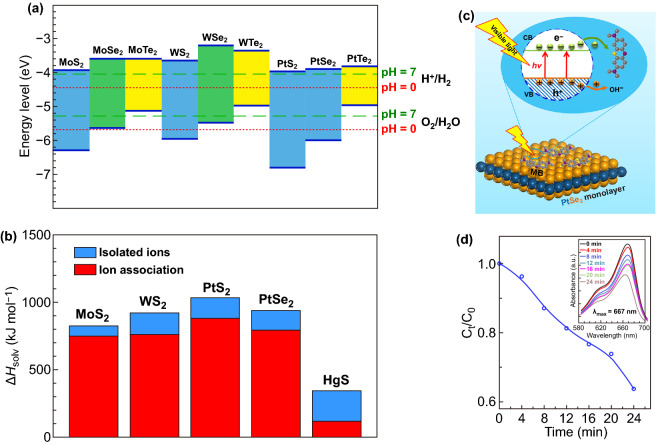
Photocatalytic properties of PtSe_2_. **a** CBM and VBM edge positions of monolayer TMDCs relative to the vacuum. **b** Comparison of the calculated solvation enthalpies (ΔHS_olv_) of monolayer PtSe_2_, PtS_2_, MoS_2_, and WS_2_ to the value of HgS. The ΔHS_olv_ of insoluble HgS is shown for comparison which shows a negligible solubility of 1.27 × 10^−27^ mol/100 g of H_2_O. Reproduced with permission [40]. Copyright 2019, American Chemical Society. **c** Schematic diagram of the photocatalytic degradation mechanism of methylene blue (MB) molecular over PtSe_2_ film. **d** Relationship between *C*_*t*_*/C*_0_ and the photocatalytic degradation time under visible light (*C*_*t*_ and *C*_0_ are the MB concentrations at time t and 0 min). The inset shows the UV–Vis absorption spectra of MB solution for every 4 min. Reproduced with permission [25]. Copyright 2015, American Chemical Society

2D materials have been widely studied as high-performance photocatalyst due to its large specific area and excellent electronic properties [185–188]. In 2013, Zhuang et al. [40] performed a systematic theoretical study on the photocatalytic performance of monolayer TMDCs by using first principles calculation. As shown in Fig. 13a, the position of CBM and VBM of some monolayer 2D TMDCs at pH = 0 and 7 is summarized. According to the calculation results, PtSe_2_, PtS_2_, MoS_2_, and WS_2_ show potential for photocatalysis. Moreover, the solvation enthalpies (ΔHS_olv_) of monolayer PtSe_2_, PtS_2_, MoS_2_, and WS_2_ are calculated, as shown in Fig. 13b. For both case of isolated and associated ions, the ΔHS_olv_ are significantly large than the value of HgS, which set as a reference. These calculation results indicate that these monolayer 2D TMDCs are insoluble and stable in aqueous solution, which make them ideal candidate for high-performance photocatalyst. The calculation results show that few-layer PtSe_2_ exhibit great potential for high-performance photocatalysis due to the sizable band gap within the visible wavelength range.

Wang et al. [25] carried out a methylene blue photocatalytic degradation experiment to evaluate the photocatalytic property of PtSe_2_ film. As shown in Fig. 13c, d, the schematic diagram of the photocatalytic degradation of methylene blue process and the time dependent photocatalytic degradation of methylene blue were evaluated by test the concentration of the methylene blue aqueous solution. It can be seen that almost 38% of methylene blue molecules are degraded in 24 min. As the PtSe_2_ catalyst absorbed a photon, an active electron–hole pair generated and the absorbed methylene blue are degraded by the high energy photon excited electrons. For comparison, the monolayer PtSe_2_ exhibits high photocatalytic degradation rate comparable with the nitrogen doped TiO_2_ nanoparticles [189].

Sun et al. [127] fabricated a PtSe_2_ film onto FTO substrate via TAC process and studied the solar-driven water splitting performance of the PtSe_2_ film. The highest photocatalytic H_2_ production rate can reach 506 mmol hm^−1^. The photocatalytic activity of the PtSe_2_/FTO thin film has no obvious decrease in ambient and acidic/alkaline solution even after aging for 1 year. Moreover, the PtSe_2_-based composite also shows high photocatalytic performance, such as PtSe_2_/graphene [130, 131] and PtSe_2_/TiO_2_/graphene [131–133].

### Hydrogen Evolution Reaction

Hydrogen has been recognized as the future energy carrier due to its ultrahigh energy density as a sustainable clean energy source [190, 191]. Experimental and theoretical efforts have indicated that 2D TMDCs materials can serve as ultrathin electrocatalysts for the hydrogen evolution reaction (HER) [38, 186, 192–195].

**Fig. 14:**
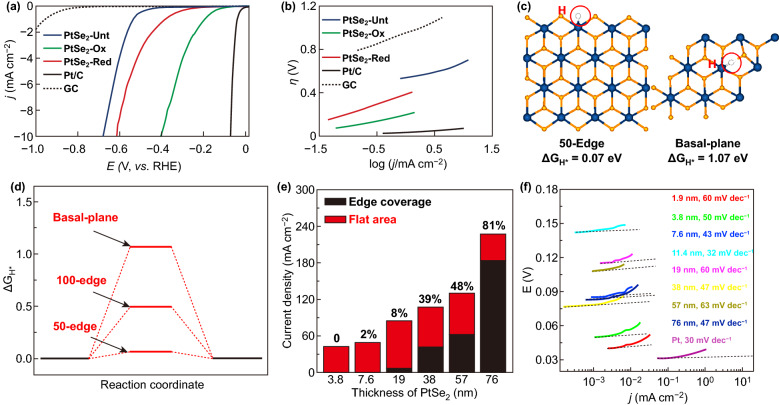
HER properties of PtSe_2_. **a** Linear sweep voltammograms versus the reversible hydrogen electrode (RHE) for HER on PtSe_2_, electrochemically oxidized PtSe_2_ and electrochemically reduced PtSe_2_. **b** Tafel plot for PtSe_2_, electrochemically oxidized PtSe_2_ and electrochemically reduced PtSe_2_. **a**, **b** Reproduced with permission [196]. Copyright 2016, John Wiley and Sons. **c** Density functional theory theoretical calculations of the H adsorption energies at the 50-edge and basal-plane of monolayer 1T-PtSe_2_. White ball: H atom, Blue ball: Pt atom, and yellow ball: Se atom. **d** Gibbs free energies (ΔG_H*_) diagram of different H adsorption states on 1T-PtSe_2_. **c, d** Reproduced with permission [197]. Copyright 2019, American Chemical Society. **e** Relationship between the current density and edge sites density on the top surface of PtSe_2_ film and the corresponding **f** Tafel plots. **e–f** Reproduced with permission [128]. Copyright 2017, Elsevier

Chia et al. [196] studied the HER electro-catalytic properties of Pt dichalcogenides by performing DFT calculations. As shown in Fig. 14a, b, the PtSe_2_ has over-potential of 0.63 eV and Tafel slop of 132 mV dec^−1^. However, the HER performance of PtSe_2_ can be further enhanced by both reduction and oxidation process. For example, the oxidized PtSe_2_ has over-potential of 0.36 eV and Tafel slop of 93 mV dec^−1^. The HER performance for PtSe_2_ is activated by both oxidation and reduction, and the oxidized and reduced PtSe_2_ exhibited better HER efficiency by a 46% and 9% decline in over-potential, respectively. Wang et al. investigated the HER performance of CVD synthesized 2H-PtSe_2_ and 1T-PtSe_2_ single crystal nanosheets. The 2H-PtSe_2_ shows the Tafel slope of 78 mV dec^−1^, which is much higher than that of 1T-PtSe_2_ (48 mV dec^−1^) [106]. Due to the semimetallic structure, the 1T-PtSe_2_ exhibits relatively higher electrochemical activity (lower Tafel slop and higher over-potential). Shi et al. also found that the monolayer or few-layer 1T-PtSe_2_ can serve as high-performance HER catalyst, and a record high HER efficiency [197]. As shown in Fig. 14c, d, the catalytic activity of monolayer 1T-PtSe_2_ was calculated by DFT to identify the electrocatalytically active sites. The calculated Gibbs free energy (Δ*G*_H*_) values of H adsorption at the 50-edge, 100-edge, and basal planes of the monolayer 1T-PtSe_2_ are 0.07, 0.50, and 1.07 eV, respectively. The relatively low Δ*G*_H*_ values for H adsorption at the edges indicate that the catalytically active sites mainly sit at the domain edges of 1T-PtSe_2_. Besides, the lower Gibbs free energy values endow the monolayer 1T-PtSe_2_ with excellent HER activity.

In 2017, Lin et al. proposed a facile strategy to synthesize edge rich PtSe_2_ film with controlled edge density and make it possible to systematic study the relationship between the edge density and the HER performance [128]. A linear relationship between the edge density and the current density on the top surface of PtSe_2_ film is established, as shown in Fig. 14e. As shown in Fig. 14f, the Tafel slope of PtSe_2_ with different thickness ranging from 32 to 63 mV dec^−1^ can be found. The current density increases with the edge density increases, which suggested that the edge density plays a key role in enhancing the HER activity of PtSe_2_.

The HER performance of PtSe_2_ has been experimentally and theoretically studied in the past few years. It has been revealed that the number of layers, edge density, and defect engineering play a key role in enhancing the HER activity of PtSe_2_ [38, 142, 196–198]. However, the relationship between the structure, electronic structure, and HER activity of 2D PtSe_2_ still is not elucidated, and the batch production of 2D metallic PtSe_2_ is still not controllable enough in experimental.

### Sensors

Sensors are a kind of integrated circuit devices that detect a specific physical parameter (gas, pressure, motion, moisture, etc.) and convert it to an electrical signal. Theoretical simulation is a very effective approach to analyze and predict gas sensing properties of 2D PtSe_2_ materials. In 2017, Sajjad et al. [199] conducted a systematically theoretical study on the absorption of various gases molecules on monolayer PtSe_2_ by using first principles calculations. The adsorption energy, relaxed height, charge density differences, and electronic structure of monolayer PtSe_2_ with absorbed CO, CO_2_, H_2_O, NH_3_, NO, and NO_2_ molecules were calculated, and the results indicate that sensors based on 2D PtSe_2_ posse superior gas detection sensitivity. Chen et al. [200] investigated the response of a simulated monolayer PtSe_2_-based gas sensor to the five types of SF6 decompositions (HF, H_2_S, SO_2_, So_2_F_2_, and SOF_2_) by using the first principles study. The sensor shows rapid and intense response to the SF6 decomposition molecular, and it could be controlled by regulating the bias voltage. Moreover, theoretical simulation suggested that the gas sensitivity of PtSe_2_ can be further enhanced by the *p*-type dopants of Ge and As [201].

**Fig. 15:**
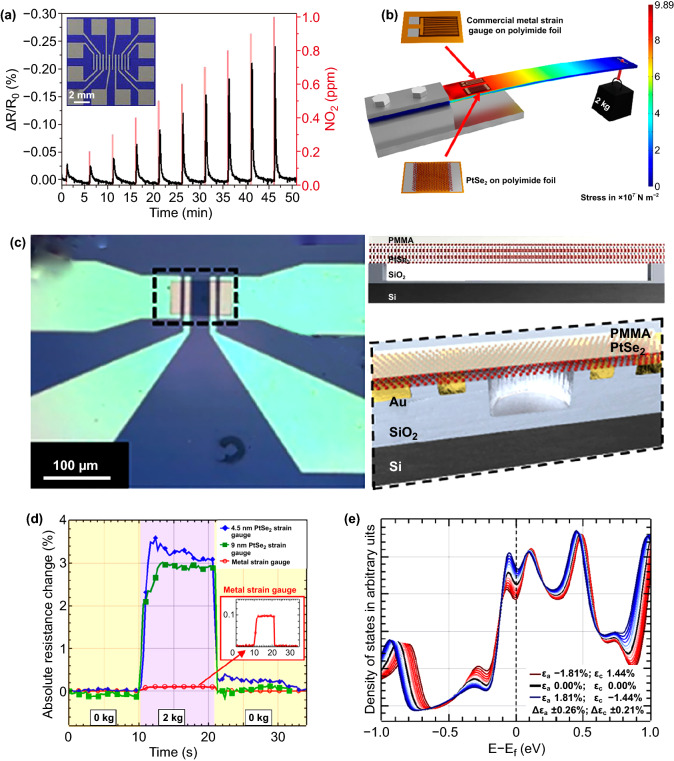
Performance of 2D PtSe_2_-based Sensors. **a** Gas sensor response of PtSe_2_ film upon periodic NO_2_ gas (0.1 to 1 ppm) exposure versus time with a bias voltage of 1 V. Red line indicates NO_2_ gas injections, and black line indicates the resistance change. Inset shows an optical microscopic image of a contacted sensor device. Reproduced with permission [112]. Copyright 2016, American Chemical Society. **b** Bending beam setup with applied PtSe_2_ film and commercially available metal strain gauges including stress simulation with the applied weight. **c** Optical microscopic image (left) of the pressure sensors with the PtSe_2_ channel across the cavity area. Structure diagram (right) of the cross section of the cavity area with suspended PtSe_2_ film. **d** Measured resistance changes during the measurement against time **e** Effect of the applied interlayer tensile strain (*ε*_a_) and compression (*ε*_c_) on the density of states close to the Fermi level. **b–e** Reproduced with permission [122]. Copyright 2018, American Chemical Society

Besides the theoretical simulation, also some experimental achievements have been reported. Figure 15a shows the PtSe_2_ film-based gas sensors and its response to periodic NO_2_ gas [112]. As the PtSe_2_ film exposed to a 100 sccm flow of NO_2_ mixture with N_2_ carrier gas, an immediately response time upon to 10 s was measured. The resistance change, transient response/recovery time as a function of NO_2_ concentration at a certain exposure time was further tested. The sensors exhibit ultrafast response/recover speed at room temperature. Moreover, 100 ppb of NO_2_ can be detected at room temperature and the theoretical limit of detection is estimated to be a few parts per billion. The detection limit, sensitivity, responses/recovery time of 2D PtSe_2_ gas sensors is much better than other 2D materials, such as graphene, MoS_2_, MoSe_2_, and MoTe_2_ [202–205].

The unique structural and electronic properties of 2D PtSe_2_ also make it a promising material for pressure sensors. As shown in Fig. 15b, c, centimeter-scale PtSe_2_ films with thickness of 4.5 and 9 nm were synthesized and used to fabricate pressure sensors [122]. The sensitivity of the PtSe_2_ film-based sensors can reach 1.05 × 10^−1^ mbar^−1^, which is much better than other low-dimensional materials-based pressure sensors [206–210]. As shown in Fig. 15d, the piezo-resistive gauge factor of PtSe_2_ film was measured by using a bending beam setup, and a negative gauge factor of − 84.8 was obtained for the PtSe_2_ film. According to the DFT calculation in Fig. 15e, an increase in DOS at Fermi level is observed for the in-plane stretching and out-of-plane compression, leading to a decrease in resistance under the applied stains and ascribe to the negative gauge factor. Moreover, Boland et al. [41] further demonstrated that the growth temperature and thickness of the PtSe_2_ film have a great effect to the performance of the PtSe_2_-based strain gauges. They found that the PtSe_2_-based pressure sensors show strong response to high frequency mechanical vibrations. By attaching a film to a speaker, a strong resistance changes of PtSe_2_/Polyimide film, with high signal-to-noise, is seen for to vibrations with frequencies of 95, 190, and 380 Hz were observed. These achievements suggest PtSe_2_ as a very promising candidate for future micro- and nanoelectromechanical systems applications.

## Conclusions and Perspectives

During the last decades, the newly emerged 2D PtSe_2_ has exhibited noticeable intrinsic nature and has experienced a remarkable development in theoretical and experimental. The most recent advances of 2D PtSe_2_ including structure (crystal structure and electronic structure), properties (phase transition, vibration spectroscopic modes, and optical properties), synthesis methods (CVD, CVT, TAC, MBE, CVT, and sol–gel solution process), and potential applications (photodetectors, mode-locked laser, field effect transistors, photocatalytic, hydrogen evolution reaction, and sensors) are reviewed in this review. Although a tremendous progress has been achieved in the past few years, there are still some remaining especially for their practical application. Here, some major perspectives on the key challenges and the potential research directions are suggested to address these issues.

1. In order to fulfill the using demands for both fundamental studies and practical applications, more efficient and controllable synthesis methods should be developed. Previous study of graphene and TMDCs has inspired us that CVD is one of the most promising methods to grow 2D materials. However, the CVD growth of 2D PtSe_2_ is still in its infancy. More compressive works about CVD should be developed to grow high quality single crystal 2D PtSe_2_ with controlled thickness, lateral size, and defects, which is prerequisite for further understanding the optoelectronic properties of PtSe_2_. Besides, in order to fulfill the demand of industrialization, highly efficient synthetic approaches should be proposed to synthesize a mass of high quality 2D PtSe_2_.
2. 2D PtSe_2_ has been theoretically predicted to be a promising candidate to fabricate high-performance electronic and optoelectronic devices [211–224]. Although some pioneer works have been reported, the performance of 2D PtSe_2_-based devices are stills much lower than theoretical prediction. Due to the layer-dependent band gap of 2D PtSe_2_, photodetector based on 2D PtSe_2_ may have excellent performance in a broadband from visible light to mid-IR. Vertical or lateral heterostructure based on 2D PtSe_2_ may bring some novel properties, which have been proved in graphene and TMDCs.
3. Theoretical studies have demonstrated that the 2D PtSe_2_ possesses excellent thermoelectric properties [67, 225, 226]. However, related experimental works are still lacking. The development of 2D PtSe_2_-based flexible film or nanostructured thermoelectric materials may provide great opportunities for fabricating highly efficient thermoelectric devices.
4. 2D Janus materials have attracted extensive attentions due to their unique structure, electronic, and optoelectronic properties [227–229]. The formation of Janus crystal structure broke the inversion and mirror symmetry, leading to an intrinsic built-in electric field. Janus monolayer 2D materials with sandwiched structure may induce remarkable influence on their carrier mobility, band gap, and optical properties. Theoretical simulations reveal that Janus monolayer PtSSe may have great potential in optoelectronics and thermal management communities. However, related experimental studies are still lacking. It is still challenging to synthesize Janus monolayer PtSSe materials and study their fundamental properties.
